# Coronary artery calcium score: current status

**DOI:** 10.1590/0100-3984.2015.0235

**Published:** 2017

**Authors:** Priscilla Ornellas Neves, Joalbo Andrade, Henry Monção

**Affiliations:** 1 Full Member of the Colégio Brasileiro de Radiologia e Diagnóstico por Imagem (CBR), MD, Radiologist at the Hospital Santa Luzia, Brasília, DF, Brazil.; 2 MD, PhD, Radiologist at the Hospital Santa Luzia, Brasília, DF, Brazil.

**Keywords:** Calcinosis/diagnosis, Cardiomyopathies/diagnosis, Tomography, X-ray computed, Cardiovascular diseases/epidemiology, Coronary artery disease/epidemiology

## Abstract

The coronary artery calcium score plays an Important role In cardiovascular risk stratification, showing a significant association with the medium- or long-term occurrence of major cardiovascular events. Here, we discuss the following: protocols for the acquisition and quantification of the coronary artery calcium score by multidetector computed tomography; the role of the coronary artery calcium score in coronary risk stratification and its comparison with other clinical scores; its indications, interpretation, and prognosis in asymptomatic patients; and its use in patients who are symptomatic or have diabetes.

## Abstract (pt)

O escore de cálcio coronariano tem papel relevante na estratificação de risco cardiovascular, apresentando significativa associação com a ocorrência de eventos cardiovasculares maiores no acompanhamento de médio e longo prazo. São discutidos: os protocolos de aquisição e quantificação por meio da tomografia computadorizada multidetectores; seu papel na estratificação de risco coronariano e relação com os demais escores clínicos; suas indicações, interpretação e prognóstico em pacientes assintomáticos; sua utilização em pacientes sintomáticos e em diabéticos.

## INTRODUCTION

Cardiovascular disease is the leading cause of death worldwide, coronary artery disease (CAD) accounting for half of all such deaths^(1)^.

At least 25% of patients experiencing nonfatal acute myocardial infarction or sudden death had no previous symptoms^(2)^. The identification of asymptomatic individuals at greater risk of experiencing future cardiovascular events is fundamental for the implementation of preventive strategies.

“Total risk scores” are very useful and should be used as the initial method of stratification, although they are able to predict only 65-80% of future cardiovascular events ^(1,2)^. The Framingham risk score is one of the most widely used^(2)^.

The characterization of coronary-artery calcification by computed tomography shows equivalence with the total coronary atherosclerosis load and the risk of cardiovascular events^(3)^.

This review on the coronary artery calcium (CAC) score addresses the following topics: acquisition and quantification protocols; stratification of coronary risk and correlation with other clinical scores; use of the CAC score in asymptomatic patients, including indications, interpretation, and prognosis; use of the CAC score in symptomatic patients; and use of the CAC score in patients with diabetes.

## ACQUISITION AND QUANTIFICATION PROTOCOLS

The CAC score was initially studied by electron beam computed tomography, a good part of the scientific literature then being based on that technique^(3)^. However, multidetector computed tomography subsequently became the modality of choice for CAC evaluation. As a consequence, electron beam computed tomography is now practically unavailable.

The determination of the CAC score by computed tomography is based on axial slices, with a thickness of 3 mm, without overlapping or gaps, limited to the cardiac region, acquired prospectively in synchrony with the electrocardiogram at a predetermined moment in the R-R interval, usually in the mid/late diastole^(1)^, without the use of intravenous contrast medium.

The effective dose of radiation is usually low, typically less than 1.5 mSv^(3)^, which is the highest effective dose recommended for use in image acquisition, according to the Society of Cardiovascular Computed Tomography^(1)^.

Calcification is identified as areas of hyperattenuation of at least 1 mm^2^-with > 130 Hounsfied units (HU) or ≥ 3 adjacent pixels^(4)^.

The main systems for the quantification of the CAC score are the Agatston method^(4)^, determination of the volume of calcium^(5)^, and determination of the calcium mass score^(6)^. The first two are the most widely used, especially the Agatston method, which is used as a reference for most population databases and publications involving risk stratification and is therefore the method most often used in clinical practice. The calcium volume score and calcium mass score have shown better reproducibility^(7)^.

**Agatston method** - The Agatston method uses the weighted sum of lesions with a density above 130 HU, multiplying the area of calcium by a factor related to maximum plaque attenuation: 130-199 HU, factor 1; 200-299 HU, factor 2; 300-399 HU, factor 3; and ≥ 400 HU, factor 4.

Therefore, the slice thickness and the interval must follow the original protocols in order to reduce the noise variation and, consequently, the maximum attenuation of the plaques, allowing the original published scores to be reproduced.

**Calcium volume score** - The calcium volume score has proven to be the most robust and reproducible method^(8)^. It is calculated by multiplying the number of voxels with calcification by the volume of each voxel, including all voxels with an attenuation > 130 HU. However, this method is particularly sensitive to the partial volume (especially in plaques with high attenuation) and subject to variability between tests, depending on the position of the plaque in the axial slice acquired.

**Relative calcium mass score** - The relative calcium mass score is calculated by multiplying the mean attenuation of the calcified plaque by the plaque volume in each image, thus reducing the variation caused by the partial volume. The absolute calcium mass score uses a correction factor based on the attenuation of water^(8)^.

## STRATIFICATION OF CORONARY RISK AND RELATIONSHIP OF THE CAC SCORE TO OTHER CLINICAL SCORES

The CAC score plays a relevant role in the stratification of cardiovascular risk. Several studies have shown that the CAC score is significantly associated with the occurrence of major cardiovascular events (all-cause mortality, cardiac mortality, and nonfatal myocardial infarction) in the medium- and long-term follow-up.

In an American College of Cardiology Foundation/American Heart Association (ACCF/AHA) consensus^(9)^, data from six large studies that collectively included 27,622 asymptomatic patients were aggregated and the relative risk of major cardiovascular events was calculated for patients with a positive CAC score and for those with a CAC score of zero. The following results were obtained:

- CAC score of 100-400-relative risk of 4.3 (95% CI:3.1-6.1);
- CAC score of 401-999-relative risk of 7.2 (95% CI:5.2-9.9);
- CAC score = 1000-relative risk of 10.8 (95% CI:4.2-27.7).

The CAC score was studied in association with other well-established traditional risk score systems, especially the Framingham risk score, showing the following advantages: independent added value in the prediction of all-cause mortality and mortality due to coronary disease in asymptomatic individuals^(9)^; and reclassification in the category of coronary artery disease risk-60% of atherosclerotic coronary events occur in patients categorized as being at low or intermediate risk according to the Framingham risk score. As an example, among patients at intermediate risk according to the Framingham risk score and with a CAC score > 300, the annual frequency of myocardial infarction or coronary death would be 2.8%, which would place them in a high risk category, the 10-year event frequency therefore being approximately 28%^(10)^.

The Framingham risk score is a simple, low-cost method of cardiovascular risk stratification that can be determined in the doctor’s office and establishes the 10-year risk of CAD. The method takes into consideration age, gender, systolic blood pressure, ratio of total cholesterol to high-density lipoprotein fraction, smoking status, and the presence or absence of diabetes.

The CAC score adds value to the Framingham risk score and to other methods, providing a substantial increase in the accuracy of the risk stratification^(1,11-13)^. It is of note that the incidence of cardiovascular events reported for patients classified as being at intermediate risk by the Framingham risk score and with an elevated CAC score is equal to or greater than that reported for patients classified as being at high risk by the Framingham risk score and with a low CAC score^(1)^.

In the United States, only 1% of women between 50 and 59 years of age and 9% of men between 60 and 69 years of age would be classified as intermediate or high risk according to the Framingham criteria. However, the incidence of events in those groups is ≤ 60% and ≤ 92%, respectively^(14)^.

The CAC score is also an independent predictor of the risk of major cardiovascular events, with demonstrated superiority over the Framingham risk score, C-reactive protein level, and carotid intima-media thickness^(11,13,15-18)^.

Various studies have used the receiver operating characteristic (ROC) curve C-statistic-also known as the area under the curve-to compare different methods of predicting cardiovascular events. The ROC curve is a graph of sensitivity (rate of true-positive results) versus specificity (rate of false-positive results) and allows two or more diagnostic tests to be compared. The area under the curve ranges from 0.5 to 1.0, values > 0.7 being indicative of satisfactory performance.

A study by Detrano et al.^(19)^, who followed 6722 patients for a mean of 3.9 years and compared clinical risk factors (age, gender, blood pressure, serum cholesterol, smoking, diabetes, family history of CAD, serum triglycerides, serum creatinine, body mass index, waist circumference, and hip circumference), alone and in combination with the CAC score, found area under the curve values of 0.79 and 0.83, respectively. Other studies^(11-13,20)^ are quoted in Table 1.

**Table 1:** Comparison of the CAC score and Framingham risk score, alone and in combination, as predictors of major cardiovascular events, based on the area under the curve.

	Sample		Follow-up		Area under the (ROC) curve*		
Study	Number of patients / age		Years (mean)		CACS	FRS	CACS + FRS
Raggi et al.^(20)^	10377		5		-	0.68 (M) / 0.67 (F)	0.72 (M) / 0.75 (F)
Greenland et al.^(12)^	1312 / > 45 years		7		-	0.63	0.68
Arad et al.^(11)^	4613 / 50-70 years		4.3		0.79	0.69	-
Becker et al.^(13)^	1726 / 57.7 ± 13.3 years		3.3		0.81	0.63	-

* Area under the (ROC) curve > 0.7: satisfactory performance. CACS, coronary artery calcium score; FRS, Framingham risk score; M, males; F, females.

## THE CAC SCORE IN ASYMPTOMATIC PATIENTS: INDICATIONS, INTERPRETATION, AND PROGNOSIS

### Indications for the use of the CAC score

The use of the CAC score in asymptomatic subjects at intermediate risk, as determined by traditional clinical stratification methods, such as the Framingham risk score, is considered appropriate/recommended with a good level of evidence by the II Guidelines of the Brazilian Society of Cardiology/Brazilian College of Radiology and Diagnostic Imaging and other international consensus statements^(18,21-25)^.

The use of the CAC score is not indicated in high-risk patients, because aggressive preventive measures would already be indicated in such patients^(1)^.

Within the group of patients classified as being at low risk, we have attempted to identify a subgroup with a significant long-term risk of a cardiovascular event, for which preventive measures should be adopted. Recent evidence has shown that a family history of premature CAD (in a male first-degree relative < 55 years of age or female first-degree relative < 65 years of age) is an independent risk factor and is associated with increased atherosclerotic burden^(1)^.

Table 2 summarizes the recommendations for the use of the CAC score in asymptomatic patients, according to the main guidelines published.

**Table 2:** Recommendation for the use of the CAC score in asymptomatic patients.

		Low risk	Low risk + family		
Authority guidelines	Low risk	+ DM	history* of early CAD	Intermediate risk	High risk
2010 ACCF/SCCT/ACR^(21)^	Inappropriate	-	Appropriate	Appropriate	Uncertain
2014 ACR^(22)^	Typically inappropriate	-	Can be appropriate	Appropriate	Typically inappropriate
2010 ACCF/AHA^(23)^	IIb	-	-	IIa	-
2012 ESC^(24)^	-	-	-	IIa	-
2014 II Diretriz da SBC/CBR^(18)^	III	IIa	IIa	I	III
2013 ACC/AHA^(25)^	IIb: If, after risk assessment, the treatment based on the decision is uncertain, evaluation with the CAC score can be				
	considered in order to define the most appropriate therapeutic strategy^†^				

DM, diabetes mellitus; CAD, coronary artery disease; ACCF, American College of Cardiology Foundation; SCCT, Society of Cardiovascular Computed Tomography; ACR, American College of Radiology; AHA, American Heart Association; ESC, European Society of Cardiology; SBC, Sociedade Brasileira de Cardiologia (Brazilian Society of Cardiology); CBR, Colégio Brasileiro de Radiologia (Brazilian College of Radiology and Diagnostic Imaging).

Classes of recommendation: Class I - Conditions for which there is conclusive evidence or, in the absence thereof, general agreement that the procedure is safe and useful/effective; Class II - Conditions for which there is conflicting evidence and/or divergence of opinion on safety, and utility/effectiveness of the procedure; Class IIa - Weight of divergences in favor of the use/effectiveness of the method. Most approve; Class IIb - Safety and utility/effectiveness less well established, with no predominance of opinions in favor. Class III - Conditions in which there is evidence, general agreement or both, that the procedure is not useful and effective, and in some conditions may even be harmful.

* First-degree male relative < 55 years of age or first-degree female relative < 65 years of age.

† After discussing with the patient, when the decision to initiate statin therapy is difficult to make in selected individuals who are not in one of the four groups benefiting from the use of statin, defined as described: atherosclerotic cardiovascular disease (ACD); primary elevation of low-density lipoprotein cholesterol (LDL C) ≥ 190 mg/dL; 40-75 years of age with diabetes and an LDL-C of 70- 189 mg/dL; and 40-75 years of age without ACD or diabetes, with an LDL-C of 70-189 mg/dL and a ≥ 7.5% estimated 10-year risk of ACD.

### Interpretation of the CAC score result

The values obtained from the CAC score can be interpreted and classified in two ways: using the absolute values with fixed cut-off points; and adjusting values for patient age, gender, and ethnicity, as well as calculating distribution percentiles in the general population through the use of several population databases, the Multi-Ethnic Study of Atherosclerosis (MESA)^(26)^ being the most widely used.

The MESA was a prospective cohort designed to investigate the prevalence, risk factors, and progression of subclinical cardiovascular disease, following 6814 initially asymptomatic patients, 45-84 years of age, including White, Black, Hispanic, and Chinese-American residents of various communities within the United States^(26)^.

The MESA demonstrated that coronary calcifications are more common in men. In the MESA sample, a score of zero was observed in nearly two thirds (62%) of the women and in 40% of the men. In terms of ethnicity, the prevalence of CAC, regardless of gender, was highest among the White subjects. Among the males, that prevalence was lowest for Black individuals, whereas it was lowest for Hispanic individuals among the females. Among the older patients (men over 70 years of age and women over 75 years of age), the prevalence of CAC, regardless of gender, was lowest for the Chinese-American individuals^(26)^.

**Figure 1:**
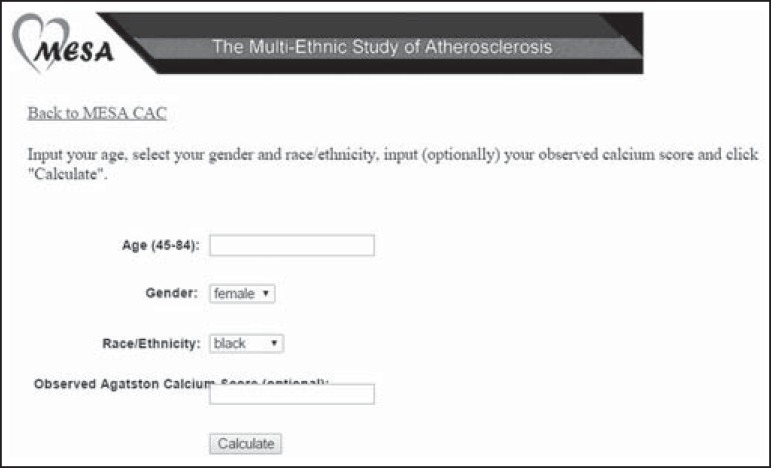
Tool for calculating the CAC score in percentiles, according to the distribution by age, gender, and ethnicity, as per the MESA.

The percentile can be calculated on the MESA website (http://www.mesa-nhlbi.org/Calcium/input.aspx) by inserting the patient CAC score (according to the Agatston method), age, gender, and ethnicity. Patients with known cardiovascular disease (acute myocardial infarction, angina, stroke, or atrial fibrillation), those using nitroglycerin, and those with a pacemaker, as well as those having undergone angioplasty, myocardial revascularization, or any other cardiac/arterial surgery, together with those under treatment for diabetes, should not be included in this analysis, given that they were not included in the MESA population (Figure 1).

**Figure 2:**
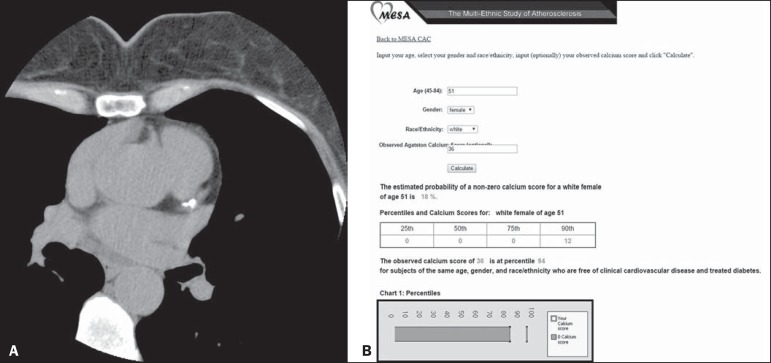
CAC score in a 51-year-old White female. **A:** Calcified plaque in the anterior descending artery. CAC score = 36 (Agatston method), consistent with discrete coronary calcification, indicating low cardiovascular risk. **B:** However, if the CAC score adjusted for age, gender, and ethnicity is used, according to the MESA, the score should be considered as being accentuated, indicating marked cardiovascular risk, because it is above the 90th percentile for this group.

**Figure 3:**
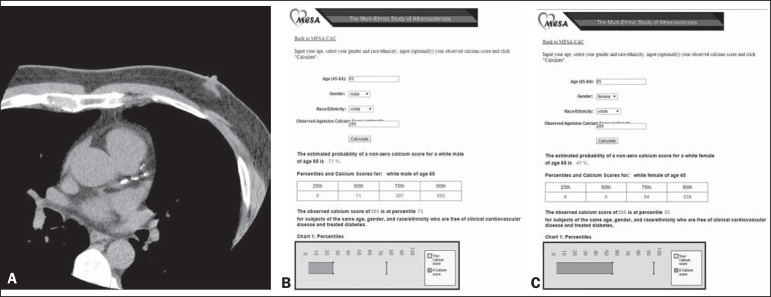
CAC score in a 65-year-old white male. **A:** Calcified plaques in the anterior descending artery, in addition to others (not shown) in the other coronary arteries. CAC score = 285 (Agatston method), consistent with moderate coronary calcification, indicating moderate cardiovascular risk. **B:** However, if the CAC score adjusted for age, gender, and ethnicity is used, according to the MESA, the calcium score should be considered discrete, indicating a low cardiovascular risk because it is below the 75th percentile for this group. **C:** If this same CAC score (Agatston 285) had been found in a woman of the same age and ethnicity, her adjusted score would be considered pronounced, indicating marked cardiovascular risk (above the 90th percentile).

The most widely used classification systems for the categorization of calcium scores-one using absolute values and one using those based on percentiles adjusted for gender, age, and ethnicity-are shown in Table 3, together with their clinical interpretation^(15,18)^. Both classification systems provide valuable prognostic information that should be included in the reports. Figures 2 and 3 illustrate examples of the use of the CAC score in two patients, showing absolute values and those based on percentiles adjusted for gender, age, and ethnicity according to the MESA.

**Table 3:** Degree of coronary artery calcification by absolute CAC scores and CAC scores adjusted for gender, age and ethnicity, with clinical interpretations

Degree of coronary	Absolute CAC score	CAC score adjusted for gender,	
artery calcification	(Agatston method)	age and ethnicity - percentile	Clinical interpretation
Absent	0	0	Very low risk of future coronary events
Discrete	1-100	≤ 75	Low risk of future coronary events; low probability of myocardial ischemia
Moderate	101-400	76-90	Increased risk of future coronary events (aggravating factor); consider reclas-
			sifying the individual as high risk
Accentuated	> 400	> 90	Increased probability of myocardial ischemia

Various studies have demonstrated the utility of CAC scores in guiding the clinical management of CAD in asymptomatic patients. The (U.S.) National Cholesterol Education Program guidelines recommend intensification of low-density lipoprotein cholesterol reduction in patients with multiple risk factors and a CAC score above the 75th percentile^(27)^. Other studies have correlated CAC scores with the use of statins and aspirin in primary prevention^(28,29)^. Table 4 summarizes some of those studies.

**Table 4:** CAC score. Prognosis and recommended treatment strategies.*

		CAC score = 0	CAC score 1-100	CAC score > 100
Population (% patients)^(28)^		56%	26%	18%
Annual frequency of events^(29)^		0.1%	0.5%	1.9%
Annual frequency of cardiovascular events^(28)^		0.4%	0.8%	2.4%
Number needed to treat (to prevent one cardiovascular event over a five year period)				
Treatment with aspirin - Number needed to treat^(28)^	FRS < 10%	2036	571*	173
		808	146*	92
Treatment with statins - Number needed to treat^(30)^	FRS ≥ 10%	549	94	24
Treatment recommendations				
	CAC score = 0	CAC score 1-100		CAC score > 100
Recommended	None	Tailored use of statins + aspirin		Statins + aspirin
Recommendation for all patients	Life style change + monitoring of cardiovascular risk factors			

* The estimated number needed to produce damage from aspirin use (one episode of major bleeding over the five year period) is 442 patients(28). Therefore, when the anticipated benefit exceeds the risk (e.g., when the FRS is ≥ 10% in patients with a calcium score of 1-100), the use of aspirin should be considered. CAC score (Agatston method). FRS, Framingham risk score.

### Prognostic value of a CAC score of zero in asymptomatic patients

Various studies have shown that asymptomatic patients with a CAC score of zero have a low risk of cardiovascular events or all-cause mortality in the medium and long term^(9)^.

A meta-analysis, published in 2009^(30)^, included 13 studies with a collective total of 29,312 patients and an average follow-up of 50 months. The authors found that, on average, a cardiovascular event occurred in 0.47% of the patients with a CAC score of 0 and in 4.14% of those with a positive score, corresponding to a relative risk of 0.15 (95% CI: 0.11-0.21; *p* < 0.001).

In a 2007 cohort study conducted by Budoff et al.^(16)^, 25,253 patients were followed for up to 12 years (mean, 6.8 years). The authors found that, among the patients with a CAC score of 0, the mortality rate was low (0.4%), confirming the low long-term risk of mortality associated with such a score.

However, there are still no recommendations to limit the use of preventive measures, such as lipid-lowering medications, if the patient is classified as being at intermediate or high risk by the traditional scores^(9,18)^.

### When should the use of the CAC score be repeated?

Some studies have demonstrated that an increase in the CAC score can have value in clinical practice to evaluate the progression of atherosclerotic plaques and the future cardiovascular risk^(1,31,32)^.

There is no well-defined method for calculating the progression of atherosclerotic plaques. The higher the CAC score is, the greater is the variability across studies^(32-34)^.

The progression of atherosclerotic plaques is overestimated when absolute values are used in patients with a high initial CAC score. If the percentage increase in relation to the initial examination is used, the progression will be overestimated in patients with a low score. For example, if a patient had a baseline CAC score of 10 and a score of 15 in the follow-up evaluation, the proportional progression would be 50%, which would correspond to a progression from 100 to 150 in a patient with a higher score^(32,33)^.

Preliminary studies have shown that an annual increase of ≥ 15% in the volume of coronary calcium would be related to a 17-fold increase in the risk of a cardiovascular event^(23)^. Currently, the most widely accepted method is the one proposed by Hokanson et al.^(34)^, who suggested a mathematical regression model, with transformation of the square root of coronary calcium volume, considering an increase ≥ 2.5 mm^3^ to be a significant degree of progression.

Some authors have suggested that the volume of calcium be included in the report for possible future comparisons. However, more prospective studies are needed, and there are as yet insufficient data to use the progression of the CAC score in clinical practice. The use of the CAC score to monitor treatment with drugs, especially statins, has been speculated. Preliminary retrospective studies and prospective cohort studies have suggested that statin use slows the progression of the CAC score. However, these results were not reproduced in randomized controlled trials^(1,32)^. Although statin therapy can reduce fibrolipid plaques, its effect on calcified plaques is questionable. Pathophysiologically, statins can promote microcalcifications in the plaques and might even increase the CAC score^(32)^. The consensus statements issued to date do not indicate that the CAC score should be determined as a method of monitoring therapeutic interventions.

Studies have shown that a follow-up examination of patients with a CAC score of zero would not be needed until four or five years after the initial examination^(28,29)^. Min et al.^(35)^ showed a progression from a CAC score of zero to a positive CAC score, the score increasing by 0.5% in the first year, 1.2% in the second year, 5.7% in the third year, 6.2% in the fourth year, and 11.6% in the fifth year, with mean time to conversion of 4.1 ± 0.9 years. The authors found that the time to conversion tended to be shorter among patients with diabetes, smokers, and individuals over 40 years of age.

## USE OF THE CAC SCORE IN SYMPTOMATIC PATIENTS

A meta-analysis based on articles published between 1990 and 2008 analyzed the CAC score in symptomatic patients, correlating it with the occurrence of cardiovascular events, the presence of significant stenosis on angiography, the diagnostic accuracy of the calcium score for myocardial ischemia, and the detection of acute coronary syndrome in the emergency room^(30)^. Those correlations will be discussed below.

### A CAC score of zero and the occurrence of cardiovascular events

On the topic of the occurrence of cardiovascular events in patients with a CAC score of zero, we identified seven studies, collectively involving 3924 patients, with an average follow-up of 42 months. On average, cardiovascular events occurred in of 1.8% of the patients with a CAC score of zero and in 8.99% of those with a positive score, corresponding to a relative risk of 0.09 (95% CI: 0.04 to 0.20; *p* < 0.001).^(30)^

Despite the small number of studies involving symptomatic patients, there is evidence that the risk of cardiovascular events is lower in individuals with a CAC score of zero. However, more studies are needed in order to determine the true role of the CAC score, along with other diagnostic methods, such as coronary computed tomography angiography and stress myocardial perfusion imaging, in symptomatic patients.

### A CAC score of zero and significant stenosis on coronary angiography

On the topic of significant stenosis on coronary angiography in patients with a CAC score of zero, we identified 18 studies, involving a collective total of 10,355 symptomatic patients undergoing catheterization due to suspected CAD or acute coronary syndrome; stenosis > 50% was observed in 56% of the patients, of whom 98% had a positive CAC score. These data, taken together, show that a positive CAC score, as a predictor of stenosis > 50%, has a sensitivity of 98%, a specificity of 40%, a negative predictive value (NPV) of 93%, and a positive predictive value (PPV) of 68%^(30)^. Based on that high NPV, some authors suggest that patients with a CAC score of zero would not require further ancillary examinations. However, other studies have demonstrated that the absence of coronary calcification is not a reliable indicator of the absence of significant luminal reduction. Two studies stand out:

- Subgroup of the CORE64 study: Gottlieb et al.^(36)^ demonstrated an NPV of 68%, concluding that a CAC score of zero does not exclude coronary disease. However, it should be borne in mind that the patients in that study had a higher pretest probability of coronary disease.
- Subgroup of the CONFIRM registry^(37)^, which included 10,037 symptomatic patients and showed coronary stenosis ≥ 50% and ≥ 70% in 3.5% and 1.4%, respectively, of the patients with a CAC score of zero.

### A CAC score of zero and myocardial ischemia in myocardial perfusion studies

On the topic of myocardial ischemia in myocardial perfusion studies in patients with a CAC score of zero, we identified eight studies, collectively involving 3717 patients undergoing stress myocardial perfusion imaging, among whom, on average, myocardial ischemia occurred in 7% of the patients with a CAC score of zero and in 13% of those with a positive score, corresponding to an odds ratio of 0.086 (95% CI: 0.024-0.0311; *p* < 0.0001). The NPV was 93%^(30)^.

### A CAC score of zero and acute coronary syndrome in the emergency room

On the topic of acute coronary syndrome in the emergency room in patients with a CAC score of zero, we identified three studies, involving a collective total of 431 patients with acute chest pain, testing negative for troponin, and with inconclusive electrocardiography results. Acute coronary syndrome was observed in only 1.1% of the patients with a CAC score of zero, a positive CAC score showing a sensitivity of 99%, a specificity of 57%, an NPV of 99%, and a PPV of 24% as a predictor of acute coronary syndrome. Because the sample analyzed was small, it was not possible to draw any conclusions regarding the role of the CAC score in the emergency room^(30)^.

The ACCF/AHA consensus^(9)^ suggested that the CAC score can be used as a filter before the indication for coronary angiography or for hospitalization of patients with chest pain, especially those with atypical symptoms.

The consensus published by The National Institute for Health and Clinical Excellence recommends that the CAC score be applied in patients with chest pain who are classified as being at low to intermediate risk. If the CAC score is zero, no other examination would be indicated; if the score is between 1 and 400, the consensus recommends coronary angiography; and if the score is > 400, coronary angiography would be indicated^(38)^.

The determination of the CAC score, in isolation, is quite limited for the evaluation of patients with suspected acute coronary syndrome. Therefore, the pre-test probability of cardiovascular events should always be given weight in the interpretation of the CAC score as a filter or tool to determine the clinical practice and to recommend other more or less invasive diagnostic methods in symptomatic individuals.

## USE OF THE CAC SCORE IN PATIENTS WITH DIABETES

Patients with diabetes present a risk of cardiovascular events similar to that of patients with a clinical history of atherosclerotic disease^(18)^.

Despite the higher cardiovascular risk and higher prevalence of ischemia on functional tests, there is no evidence so far that routine screening for silent ischemia reduces mortality in this group of patients.

The presence of any degree of CAC in patients with diabetes mellitus translates to a higher risk of all-cause mortality than in patients without diabetes^(33)^.

Kramer et al.^(39)^ reviewed eight studies involving a collective total of 6,521 patients and found that individuals with diabetes and a CAC score < 10 were 6.8 times less susceptible to all-cause mortality and cardiovascular events, as well as to cardiovascular events alone, than were those with diabetes and a CAC score > 10. A CAC score > 10 was associated with an increased risk of mortality and cardiovascular events in such individuals, with high sensitivity and low specificity^(39)^.

Several international guidelines have shown that screening for silent ischemia is not warranted in patients with diabetes and a CAC score < 100, although it is recommended in those with a CAC score > 400 ^(18)^.

The CAC score allows better stratification of cardiovascular risk in the heterogeneous population of individuals with diabetes, allowing identification of the individuals at the greatest risk, who could benefit from screening for silent ischemia and from more aggressive clinical treatment.

The absence of CAC indicates a low risk of death in the short term, and the annual mortality rate is similar to that of individuals without diabetes^(18,33)^.

## CONCLUSION

The CAC score is an independent marker of risk for cardiac events, cardiac mortality, and all-cause mortality. In addition, it provides additional prognostic information to other cardiovascular risk markers.

The well-established indications for the use of the CAC score include stratification of global cardiovascular risk for asymptomatic patients: intermediate risk based on the Framingham risk score (class I); low risk based on a family history of early CAD (class IIa); and low-risk patients with diabetes (class IIa).

In symptomatic patients, the pre-test probability should always be given weight in the interpretation of the CAC score as a filter or tool to indicate the best method to facilitate the diagnosis. Therefore, the use of the CAC score alone is limited in symptomatic patients.

In patients with diabetes, the CAC score helps identify the individuals most at risk, who could benefit from screening for silent ischemia and from more aggressive clinical treatment.
